# Using Sentinel Plots to Monitor for Changes in Thrips Susceptibility to MON 88702 Cotton Containing the Cry51Aa2.834_16 *Bt* Protein

**DOI:** 10.3390/insects14060497

**Published:** 2023-05-27

**Authors:** Ashley D. Yates-Stewart, Benjamin T. Yorke, Alan Willse, Jennifer Fridley, Graham P. Head

**Affiliations:** Bayer Crop Science, Chesterfield, MO 63017, USAalan.willse@bayer.com (A.W.); graham.head@bayer.com (G.P.H.)

**Keywords:** ThryvOn cotton, MON 88702, tobacco thrips, western flower thrips, *Bacillus thuringiensis*, resistance monitoring

## Abstract

**Simple Summary:**

Transgenic *Bt* crops are important tools for growers to manage insect pests, but their use is threatened by the evolution of insect resistance, and monitoring programs are essential in detecting and responding to resistance. For *Bt* products in which insect control is not complete (“non-high-dose crops”), resistance monitoring is challenging, because insects and insect damage will be present even without resistance. Given these challenges, “sentinel plots” (designated monitoring plots) consisting of *Bt* and non-*Bt* control plots have been used to monitor for insect resistance to non-high-dose *Bt* crops by assessing changes in the efficacy of a *Bt* crop over time relative to a non-*Bt* control. We used this approach for ThryvOn™ cotton, a new non-high-dose *Bt* product targeting two sucking pest types—*Lygus* and thrips—and report here on the thrips monitoring program. Monitoring for insect resistance over time requires knowledge of the baseline susceptibility, which is the initial assessment of the insect population response to a given *Bt* crop prior to its widespread adoption. To characterize the baseline susceptibility of thrips to ThryvOn, we tested several approaches and found that the number of immature thrips on ThryvOn relative to the control cotton best characterized the efficacy of the trait.

**Abstract:**

Transgenic *Bt* crops are important tools for growers to manage insect pests, but their durability is threatened by the evolution of insect resistance. Implementing a resistance monitoring program is essential to detect and mitigate resistance. For non-high-dose *Bt* crops, resistance monitoring is challenging, because insect control is not complete, so targeted insects and insect damage will be present even without resistance. Given these challenges, sentinel plots have been used to monitor for insect resistance to non-high-dose crops by assessing changes in the efficacy of a *Bt* crop over time relative to a non-*Bt* control. We optimized a sentinel plot resistance monitoring approach for MON 88702 ThryvOn™ cotton, a new non-high-dose *Bt* product targeting two sucking pest taxa—*Lygus* (*L. lineolaris* and *L. hesperus*) and thrips (*Frankliniella fusca* and *F. occidentalis*)—and report here on the thrips monitoring methods and results. Quantifying thrips immatures was the best metric to characterize the impact of the trait, with at least a 40–60% average reduction of thrips immatures on ThryvOn relative to the control cotton at all sites with higher thrips densities. These data can be used within a ThryvOn resistance monitoring program and represent a case study for establishing a resistance monitoring approach for a non-high-dose trait product.

## 1. Introduction

In agricultural systems, various integrated pest management (IPM) strategies are used to protect crops against insect pests, including the use of crops expressing transgenic insecticidal proteins derived from *Bacillus thuringiensis* (*Bt*) [1,2]. In the United States, *Bt* crops have historically targeted lepidopteran and coleopteran pests of corn and cotton [3]. A new *Bt* cotton product, Bollgard® 3 ThryvOn™ cotton with XtendFlex® technology (hereafter referred to as “ThryvOn”, which contains the event MON 88702), offers growers an additional tool to manage *Lygus* and thrips pests, key sucking pests of cotton in the Southern United States [4,5,6,7,8]. While *Bt* crops can be very effective tools against insect pests, their durability is threatened by the evolution of insect resistance [9,10]. To delay the evolution of insect resistance, various insect resistance management (IRM) strategies are employed before and after a *Bt* crop is commercialized and planted on broad acres [3], and a resistance monitoring program is implemented to assess the changes in pest susceptibility to the product over time [11,12,13]. Monitoring pest susceptibility over time requires knowledge of the baseline susceptibility, which is the initial assessment of the insect population response to the *Bt* crop prior to its widespread adoption [14].

The resistance monitoring strategies used to assess pest susceptibility to a *Bt* crop over time depend on various factors, including whether the insecticidal protein in the *Bt* crop is a high dose (i.e., the *Bt* protein expression level is sufficiently high to kill insects with heterozygous resistance genes [15]) or non-high dose against the target pest [3]. For high-dose products, any level of injury from the target pest is considered unexpected and warrants additional investigation. This makes the early detection of resistance feasible for high-dose products, for example, through collections of insect populations from areas with elevated resistance risks (areas with high product adoption and high pest pressure) and measurements of their susceptibility to *Bt* proteins using laboratory-based bioassays [11]. Any potentially resistant populations can then be mitigated as quickly as possible in the field to prevent the spread of resistance. This strategy has been used successfully to monitor for shifts in susceptibility in the more *Bt*-sensitive lepidopteran target pests of *Bt* corn and *Bt* cotton, including the European corn borer (*Ostrinia nubilalis* (Hübner)) and tobacco budworm (*Chloridea virescens* (Fabricius)) [14]. An additional method for monitoring resistance in pests of high-dose products includes investigating reports of unexpected injury in commercial *Bt* fields.

In contrast, there are challenges associated with resistance monitoring for non-high-dose products, because target pest control is not complete; some level of insect infestation and plant damage is expected [11]. Resistance detection in commercial fields is then further complicated by insecticide applications that are used by farmers to address the surviving insects and the damage they cause. To enable easier detection of unexpected levels of pest survival and plant damage that could reflect resistance, sentinel plots have been used to monitor for resistance to non-high-dose products. Sentinel plots are designated monitoring plots consisting of *Bt* and non-*Bt* control plots used to monitor for insect resistance to non-high-dose *Bt* crops by assessing changes in the efficacy of a *Bt* crop over time relative to a non-*Bt* control. For example, sweet corn sentinel plots have been used to monitor for resistance to Cry1 and Cry2 *Bt* proteins in corn earworms (*Helicoverpa zea* (Boddie)). *Bt* sweet corn plots were planted alongside non-*Bt* sweet corn plots, and the ear damage in *Bt* relative to non-*Bt* was assessed and compared to the baseline assessments to identify any potential resistance [16,17].

ThryvOn cotton contains the Cry51Aa2.834_16 *Bt* protein (later renamed as Mpp51Aa2.834_16 [18]) and is a single mode of action, non-high-dose product that targets *Lygus* and thrips species [4]. ThryvOn is the first *Bt* crop targeting any sucking pest, and it causes incomplete neonate mortality for *Lygus* species (*Lygus lineolaris* (Palisot de Beauvois) and *Lygus hesperus* Knight [6]) and oviposition reduction for thrips (65% and 85% reduction in *Frankliniella fusca* (Hinds) and *Frankliniella occidentalis* (Pergande) oviposition, respectively [5]). Once considered secondary pests of cotton, *Lygus* and thrips are now among the most important U.S. cotton pests. *Lygus* species feed on squares and small bolls, costing U.S. growers >200 million USD annually (2021 data) in yield loss and management practices [19]. Thrips are the most important early-season pest in cotton, capable of causing a yield loss up to 50% [20] and costing U.S. growers >60 million USD annually (2021 data) [19]. ThryvOn cotton will therefore be a critical IPM tool to help growers manage these sucking insect pests.

Here, we present ThryvOn cotton as a case study for establishing an efficient and effective resistance monitoring approach for a non-high-dose *Bt* product, focusing on thrips for simplicity. Consistent with resistance monitoring approaches for other non-high-dose products, such as *Bt* corn and cotton containing Cry 1 and Cry2 proteins against corn earworms [16,17], we use a sentinel plot approach for ThryvOn. One key objective was to adapt this sentinel plot approach to the thrips species targeted by ThryvOn, because these sucking pests are less well understood than other insect pests with respect to their biology and their response to *Bt* crops and proteins. We explore different sampling methods to best characterize the baseline susceptibility of the targeted thrips species to ThryvOn cotton prior to its widespread adoption, including measuring insect damage and obtaining insect counts (immatures and adults). Additionally, we discuss how the resulting baseline susceptibility data can be used in sentinel plots within a resistance monitoring program to identify unexpected injury and/or less-than-expected control in this non-high-dose system.

## 2. Materials and Methods

### 2.1. Sentinel Plots

Academic cooperators generated baseline susceptibility data for *Lygus* (order Hemiptera) and thrips (order Thysanoptera) species to ThryvOn cotton from 10 and 8 sentinel plot locations in 2021 and 2022, respectively (Table 1). These locations were selected based on an elevated resistance risk due to the historically high insect pressure and/or expected high adoption of ThryvOn. One academic cooperator from the sentinel plot location in Arizona (site 1AZ) generated baseline susceptibility data to ThryvOn for the Western target pests: Western tarnished plant bug (*Lygus hesperus*) and Western flower thrips (*Frankliniella occidentalis*). The remaining academic cooperators throughout the Midsouth and Southeast generated baseline susceptibility data from sentinel sites for the target pests found primarily throughout the Eastern U.S. cotton belt: tarnished plant bugs (*Lygus lineolaris*) and tobacco thrips (*Frankliniella fusca*). Baseline susceptibility data for the thrips species are presented here, and as tobacco thrips are the dominant thrips species found in cotton throughout the Southern and Eastern cotton belt [21], tobacco thrips is the main focus of this manuscript. The *Lygus* methodology and results will not be discussed.

Previously, it was demonstrated that the Western pests (*L. hesperus* and *F. occidentalis*) have less resistance risk than the other target pests (*L. lineolaris* and *F. fusca*), in part due to the high amounts of effective refuge present where these pests exist [22]. In particular, alfalfa is a highly productive host of both Western pest species [23,24]. Therefore, for the purposes of generating baseline susceptibility data and to inform future resistance monitoring efforts, the majority of the annual sentinel plots were placed in areas where the resistance risk was expected to be greater based on the available refuge [22] and historic pest pressure [19] (i.e., *L. lineolaris* and *F. fusca* in the Midsouth and Southeast) and documented development of resistance to insecticides [25,26,27]. Locations such as Texas and parts of the Southeast (i.e., Alabama and Georgia) were not included due to their historically low pest pressure [19].

To characterize the baseline susceptibility, the ThryvOn mode of action was considered when developing the sentinel plot protocol: ThryvOn causes neonate mortality for *Lygus* species and a reduction in oviposition for thrips species [5]. However, as the level of control is not complete for any of the target pests [6], some level of insect infestation and damage to ThryvOn cotton was expected. Insect counts and insect damage ratings were taken from sentinel plots to characterize the baseline susceptibility of target pests to ThryvOn cotton and establish a metric that could be used in identifying unexpected injuries and triggering a remedial action plan in the future.

The sentinel plots consisted of nonreplicated single blocks of ThryvOn (Bollgard^®^ 3 ThryvOn™ cotton with XtendFlex^®^ Technology) and adjacent control/non-ThryvOn (Bollgard II^®^ XtendFlex^®^ cotton), targeting roughly a half-acre square for each block to simulate a large-scale commercial field (Bollgard II and Bollgard 3 are registered trademarks of Bayer CropScience LP). All seeds were treated with Acceleron^®^ “Basic” seed treatment, which does not have insecticidal activity. At the time of this study, Bollgard^®^ 3 ThryvOn™ cotton with XtendFlex^®^ Technology did not have full approval in certain import markets and was handled according to the Bayer Crop Science stewarded material requirements. Appropriate stewarded material requirements, including isolation methods, were used for the field releases in this study.

The insect counts and damage data at most sentinel sites were taken on a transect, with the first sample beginning near the edge of the field and the final sample being taken near the center of the half-acre block for a total of 10 samples per sampling period in 2021. The transect design was implemented to capture any potential edge effects that may have occurred. For thrips, data were generated for early-season cotton at two sampling times for most sites, targeting the 1st–2nd and 3rd–4th true-leaf stages, as thrips are early-season pests of cotton. The thrips counts were reported as both the number of immatures (i.e., non-adults) and the number of adults per 5 plants for 10 samples (*n* = 10) per time point in the ThryvOn and control plots. Thrips were counted by placing plants in an alcohol or soapy water solution and washing and or filtering insects as described [21]. The species of adult thrips were identified when possible, and where this was noted, the predominant species was confirmed as described in Table 1. Thrips damage ratings were reported per row and were based on a 0–5 rating scale [28], where 0 indicates no damage, intermediate numbers are reflective of increasing levels of damage and the curling of true leaves, and 5 indicates severe stunting and/or plant death.

In 2022, the sentinel plots were implemented with some minor modifications compared to 2021. A few sentinel plot locations changed (Table 1) and damage ratings were discontinued, because the thrips counts proved more informative than damage ratings for characterizing the baseline susceptibility. Therefore, the number of thrips count samples in 2022 was doubled (to *n* = 20) per sampling.

### 2.2. Statistical Analysis

Thrips data from sentinel plots were collected at two time points per site per year, and the counts were totaled across both time points before analysis. Quasi-Poisson regression was performed on the total (cumulative across the two time points) insect counts separately for each population (i.e., each site–year combination) to estimate the relative counts of immatures and adults, independently, on ThryvOn compared to the control cotton (ThryvOn effect) and to estimate the insect pressure at each location, defined by insect counts in the control plots.

Quasi-Poisson regression was performed using the glm function in R [29]. Poisson regression is commonly used to model count data, such as egg counts and insect counts; for these data, Gaussian-based linear models are often inappropriate, because the variance increases with the mean, violating the assumptions of linear models. For biological data, it is often the case that the variance is even greater than that predicted by Poisson models, a phenomenon called overdispersion. Quasi-Poisson regression is a generalization of Poisson regression that accounts for overdispersion; specifically, when data are overdispersed, quasi-Poisson regression will produce wider confidence intervals for the parameter estimates than Poisson regression.

For damage data, the mean and standard errors of thrips damage ratings were plotted as a function of the collection time to compare the effects of ThryvOn and the control cotton.

## 3. Results

Thrips counts (immatures and adults) and insect damage ratings were obtained from sentinel plots, as our objective was to establish a metric to best characterize the baseline susceptibility of thrips to ThryvOn cotton. These baseline data can be used in the future to develop a method to identify unexpected injury and/or less-than-expected control of ThryvOn cotton, which may indicate suspected resistance.

### 3.1. Thrips Immature Counts

**Figure 1:**
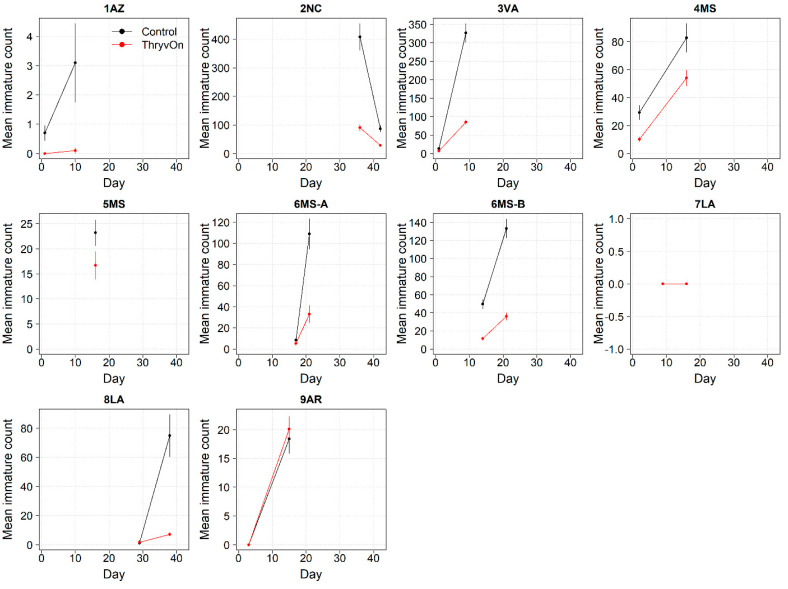
2021 immature thrips counts from sentinel plots for two sampling time points for ThryvOn (red line) and the control (black line). Data are shown as the means and standard errors per 5 plants. The 7LA site had a mean of 0 thrips for both treatments at both time points. Day 0 represents the first sample taken across all locations in 2021. The remaining days across all locations in 2021 are relative to Day 0.

**Figure 2:**
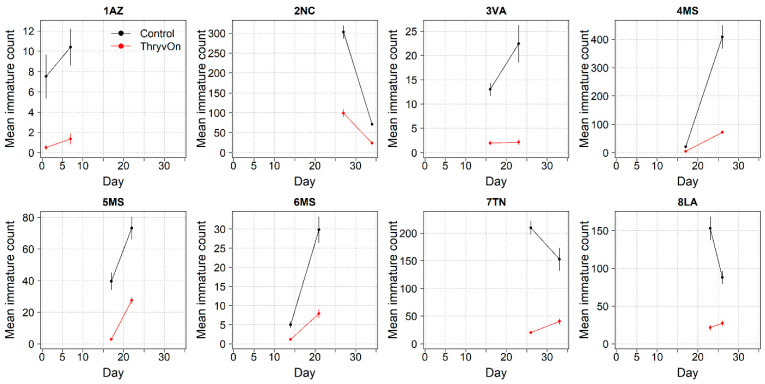
2022 immature thrips counts and standard errors from sentinel plots for two sampling time points for ThryvOn (red line) and the control (black line). Data are shown as the means and standard errors per 5 plants. Day 0 represents the first sample taken across all locations in 2022. The remaining days across all locations in 2022 are relative to Day 0.

**Figure 3:**
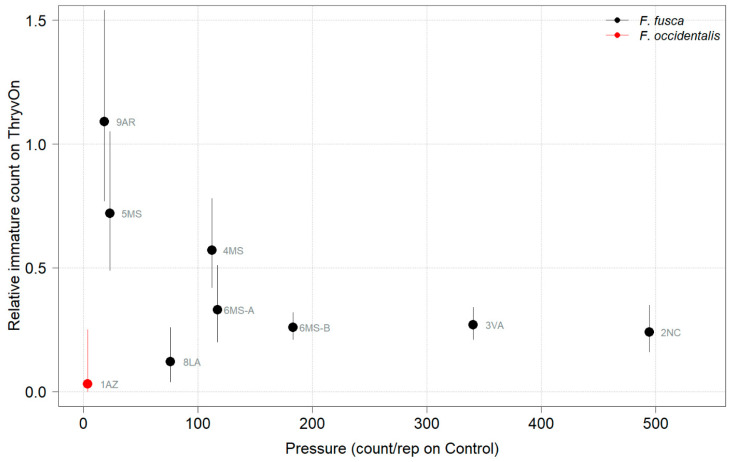
2021 thrips immature counts on ThryvOn relative to the control cotton as a function of the pressure (abundance on the control plots) generated from sentinel plots for each site. Black bars indicate *F. fusca*, and red bars indicate *F. occidentalis*. At 7LA, a mean of 0 thrips was observed in both treatments at both time points, so this site was excluded. A mean of 1 indicates no difference between ThryvOn and the control, and a mean of 0 indicates a 100% reduction of thrips on ThryvOn relative to the control cotton. Data are shown as the means with 95% confidence intervals based on quasi-Poisson regression.

**Figure 4:**
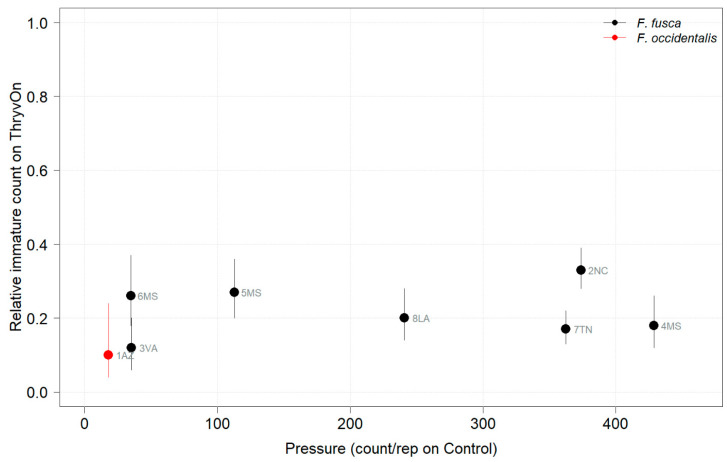
2022 thrips immature counts on ThryvOn relative to the control cotton as a function of pressure (abundance on the control plots) generated from sentinel plots for each site. Black bars indicate *F. fusca*, and red bars indicate *F. occidentalis*. A mean of 1 indicates no difference between ThryvOn and the control, and a mean of 0 indicates a 100% reduction of thrips on ThryvOn relative to the control cotton. Data are shown as the means with 95% confidence intervals based on quasi-Poisson regression.

Thrips data from sentinel plots were collected at two time points, and thrips pressure tended to be higher (as indicated by the higher control count) at a single time point for most sites (Figure 1 and Figure 2), indicating the relatively short duration of early-season thrips infestation. At most of the sites, the second time point (three to four true leaves) captured the bulk of the infestation over at least one year, as indicated by the control counts, except for the 2NC location, where the peak infestation occurred at the first collection (i.e., at one to two true leaves) in both 2021 and 2022 (Figure 1 and Figure 2). The cumulative effect of ThryvOn on the insect counts was analyzed over both time points for each year (Figure 3 and Figure 4). ThryvOn is non-high dose against thrips, and, as some level of insect survival should be expected on ThryvOn, the baseline susceptibility data are presented as the immature thrips counts on ThryvOn relative to the counts on the control plots. On the y-axis (relative number of immature thrips on ThryvOn), a mean of 1 indicates no difference between ThryvOn and the control, and a mean of 0 indicates a 100% reduction of thrips on ThryvOn relative to the control cotton. These values are plotted as a function of the overall pressure for each site (i.e., cumulative immature counts/replicate in the control plots) (Figure 3 and Figure 4). 

In 2021, the mean percent reduction of immatures on ThryvOn relative to the control cotton was >40% (i.e., relative immature count >0.6) at locations with higher insect pressure (defined as ≥50 immatures/replicate in the control plots) (Figure 3). At locations with lower insect pressure (<50 immatures/replicate in the control plots), the percent reduction of immatures on ThryvOn relative to the control was highly variable, ranging from 0% (site 9AR) to 97% (site 1AZ) (Figure 3). The locations with the highest pest pressure—6MS-B, 3VA, and 2NC (mean peak infestation in the non-ThryvOn control of 133, 327, and 408 immature counts, respectively; Figure 1)—also had levels of control >70%. Some of the variability observed at locations with lower levels of background pressure, such as 9AR and 5MS (mean peak infestation in the non-ThryvOn control of 18 and 23 immature counts, respectively; Figure 1) may be attributed to the sampling variation, so, in 2022, the number of thrips count samples was doubled, with two subsamples taken at each of the 10 locations along the transect toward the center of the field (*n* = 20).

**Figure 5:**
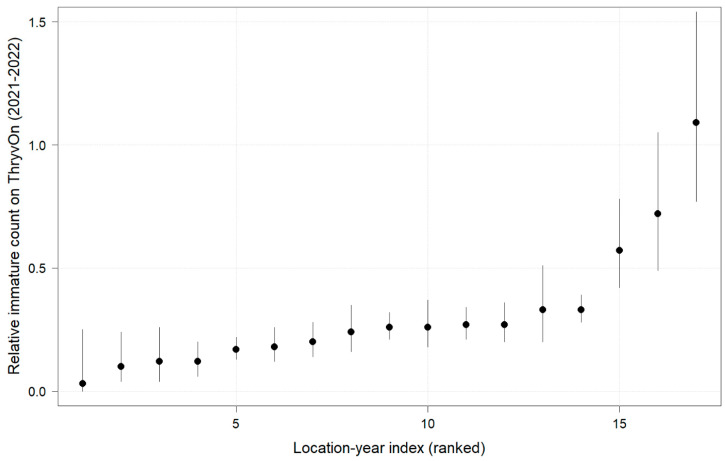
2021 and 2022 thrips immature counts on ThryvOn relative to the control cotton per sentinel plot location, ranked by relative survival of immature thrips on ThryvOn. At 7LA, a mean of 0 thrips was observed in both treatments at both time points in 2021, so this site was excluded. A mean of 1 indicates no difference between ThryvOn and the control, and a mean of 0 indicates a 100% reduction of thrips on ThryvOn relative to the control cotton. Data are shown as the means with 95% confidence intervals based on quasi-Poisson regression.

In 2022, the percent reduction of immatures on ThryvOn relative to the control was much less variable, even at locations with lower insect pressure, than in 2021 (Figure 4). Sites with the highest levels of infestation, including 4MS and 2NC (peak infestation in the non-ThryvOn control of 408 and 303 average immatures, respectively; Figure 2) showed levels of relative reduction in the immature counts comparable to those at the sites with less insect pressure, such as 3VA and 6MS (mean peak infestation in the non-ThryvOn control of <30 immatures; Figure 2). Despite varying the insect pressure across the sites, there was at least a 60% average reduction of thrips immatures on ThryvOn relative to the control cotton. The relative survival of immatures on ThryvOn relative to the control cotton across both years is shown in Figure 5; such distributions might be useful in determining a trigger threshold for potential susceptibility (see Section 4).

### 3.2. Thrips Adult Counts

**Figure 6:**
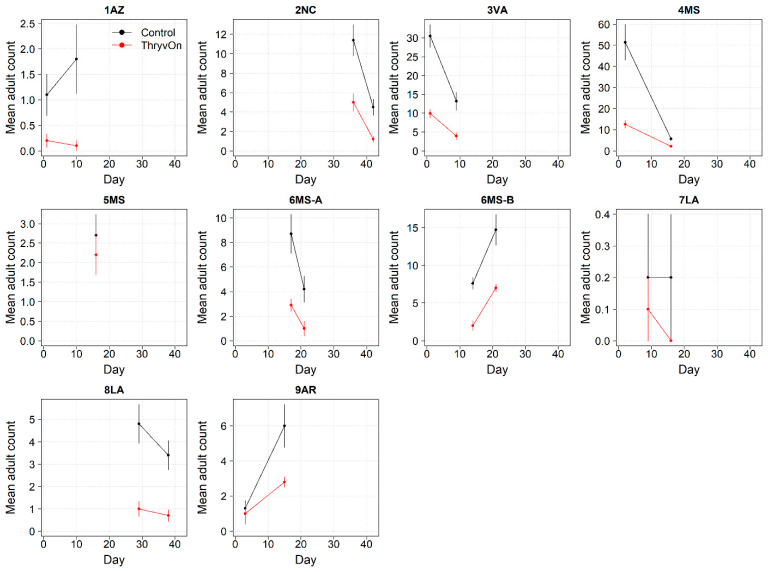
2021 adult thrips counts and standard errors from sentinel plots for two sampling time points for ThryvOn (red line) and the control (black line). Data are shown as the means and standard errors per 5 plants. Day 0 represents the first sample taken across all locations in 2021. The remaining days across all locations in 2021 are relative to Day 0.

**Figure 7:**
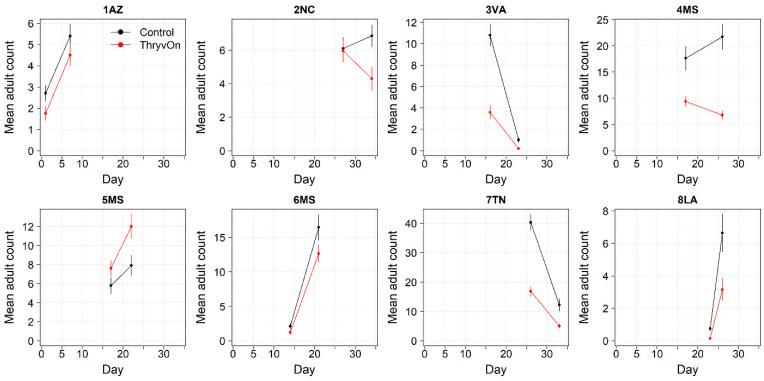
2022 adult thrips counts and standard errors from sentinel plots for two sampling time points for ThryvOn (red line) and the control (black line). Data are shown as the means and standard errors per 5 plants. Day 0 represents the first sample taken across all locations in 2022. The remaining days across all locations in 2022 are relative to Day 0.

**Figure 8:**
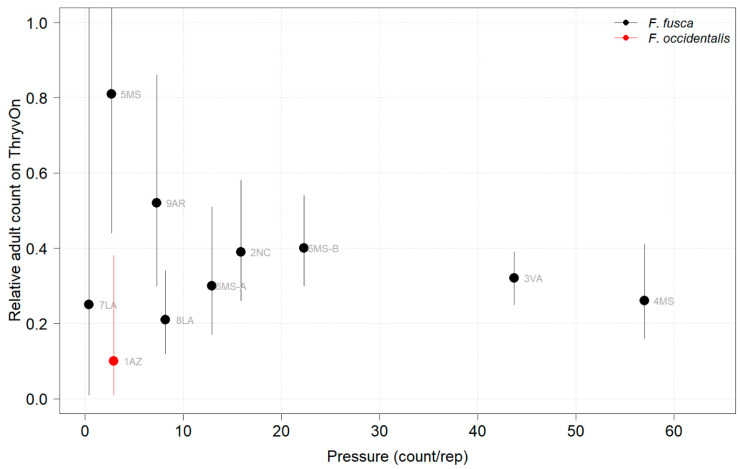
2021 thrips adult counts on ThryvOn relative to the control cotton as a function of pressure (abundance in control plots) generated from sentinel plots for each site. Black bars indicate *F. fusca*, and the red bar indicates *F. occidentalis*. A mean of 1 indicates no difference between ThryvOn and the control, and a mean of 0 indicates a 100% reduction of thrips on ThryvOn relative to the control cotton. Data are shown as the means with 95% confidence intervals based on quasi-Poisson regression.

**Figure 9:**
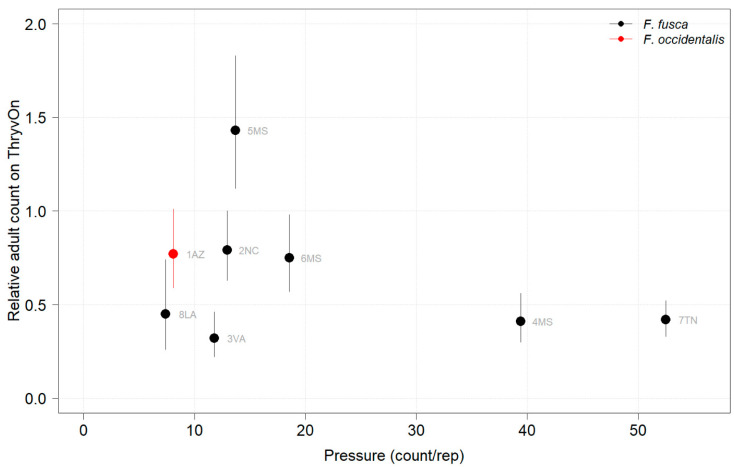
2022 thrips adult counts on ThryvOn relative to the control cotton as a function of pressure (abundance on control plots) generated from sentinel plots for each site. Black bars indicate *F. fusca*, and the red bar indicates *F. occidentalis*. A mean of 1 indicates no difference between ThryvOn and the control, and a mean of 0 indicates a 100% reduction of thrips on ThryvOn relative to the control cotton. Data are shown as the means with 95% confidence intervals based on quasi-Poisson regression.

Thrips adults were identified to the species at five Midsouth and Eastern locations in 2021 and 2022, and tobacco thrips comprised ≥ 80% of thrips at those locations. In general, the overall pressure of thrips adults at both time points in both years (Figure 6 and Figure 7) was much less than that of thrips immatures (Figure 1 and Figure 2). We expected the level of adult thrips abundance to vary greatly by location due to environmental factors, the lack of control of adults by ThryvOn, and their ability to move greater distances (i.e., between fields) at this life stage. The baseline susceptibility data are presented as the cumulative ThryvOn effect on adult thrips counts relative to the counts on the control plots (as was done for immatures), plotted as a function of the overall pressure for each site (i.e., cumulative counts/replicate in control plots) (Figure 8 and Figure 9).

In 2021, at locations with lower insect pressure (i.e., less than five adults/replicate in the control plots) (Figure 8), the reduction of adults on ThryvOn relative to the control cotton was variable (i.e., very large confidence intervals, sometimes overlapping the relative control of one). This includes sites 7LA, 1AZ, and 5MS, where the peak infestation of thrips adults on the non-ThryvOn was 0.2, 1.8, and 2.7 average individuals per sample, respectively (Figure 6). This variation at locations with lower pressure also occurred with immature thrips (Figure 3 and Figure 4). In 2022, despite doubling the thrips count samples from 2021, a reduction of adults on ThryvOn relative to the control cotton was not observed at every site (i.e., 5MS; Figure 9). A consistent ThryvOn effect on adults relative to the control cotton was not observed (Figure 8 and Figure 9), indicating that adult counts are an ineffective means of tracking the trait effect over time.

### 3.3. Thrips Damage Ratings

In addition to insect counts, insect damage ratings were obtained from sentinel plots in 2021, as our objective was to establish a metric (using either or both of these measurements) to best characterize the baseline susceptibility of target pests to ThryvOn cotton.

**Figure 10:**
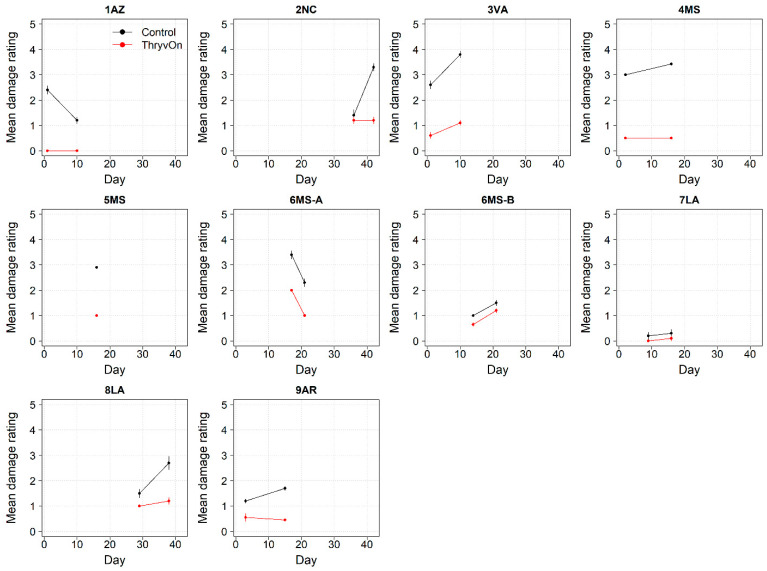
Thrips damage ratings generated from 2021 sentinel plots for each site over time for ThryvOn (red line) and the control (black line). A 0 indicates no damage, and 5 indicates severe stunting/plant death [28]. Data are shown as the means and standard errors. Day 0 represents the first sample taken across all locations in 2021. The remaining days across all locations in 2021 are relative to Day 0.

The ThryvOn damage ratings (0–5 scale) ranged from 0 to 2 (Figure 10), despite various levels of insect pressure (Figure 1, Figure 2, Figure 6 and Figure 7). In all cases, the control plots had greater damage than the ThryvOn plots, with means ranging from 0.2 to 3.8. However, as immature thrips counts were more informative than damage for characterizing the baseline susceptibility data (see Section 4), the damage ratings were not scored in 2022.

## 4. Discussion

Transgenic *Bt* crops are important tools for growers to manage insect pests, and implementing resistance monitoring programs is essential to ensuring their efficacy. Here, we optimized a resistance monitoring approach for ThryvOn cotton, a newly commercialized *Bt* product targeting *Lygus* and thrips pests [4,5,6,7]. As ThryvOn is a non-high-dose product, insect pressure and damage are to be expected, presenting challenges for characterizing the baseline susceptibility and defining a resistance monitoring strategy. Additionally, ThryvOn targets sucking insects, which are less understood than other insect pests with respect to their biology and interactions with the *Bt* trait. Given these unique challenges, we explored various methods to optimize the resistance monitoring approach to assess the impact of the trait and characterize the baseline susceptibility, focusing on tobacco thrips.

To determine the most appropriate method to characterize the baseline susceptibility, we obtained both insect counts and insect damage ratings. Insect damage ratings have been used to monitor *Bt* efficacy and insect resistance in other non-high-dose systems, including the efficacy of Cry1 and Cry2 *Bt* proteins against *H. zea* in corn [16,17]. These damage ratings include documenting the percentage of *Bt* corn ears damaged by *H. zea* and the amount of damage to *Bt* corn ears relative to non-*Bt*. For thrips, the damage to cotton seedlings is characterized by leaves that wrinkle and can have a silvery appearance or damaged meristem [20]. As such, insect damage ratings for thrips are conducted on a visual 0–5 scale that reflects an increasing damage severity [28]. Challenges for using thrips damage to characterize the baseline susceptibility to ThryvOn are that the ratings scale is more subjective and less quantitative than that for ear damage in corn, damage symptoms from thrips can be confused with damage caused by environmental conditions (such as sandblasting [30]), and, because this is a non-high-dose system, damage will typically be present. Further, insect damage ratings are ultimately tied to the presence of insects, which was a better and more direct metric to estimate the impact of the ThryvOn trait on the sentinel plots. Given these results and challenges, we did not utilize thrips damage ratings to characterize the baseline susceptibility, and we discontinued the damage ratings in 2022. It should be noted that damage ratings will continue to be integral to maintenance and decision making in commercial fields, and the methods proposed here are specifically applicable to detecting resistant populations in sentinel plots.

We also obtained insect counts of both immatures and adults to optimize the resistance monitoring approach. The overall pressure of thrips adults during both years was much less than that of thrips immatures. This is not unexpected, as adult thrips are capable of dispersing [31,32] and may be moving in and out of the plots, making it challenging to quantify the adults. Additionally, ThryvOn does not impact the thrips adult life stage but, rather, reduces oviposition [5], so it is logical that assessing the adult life stage is not an accurate characterization of the trait impact. Further, immature counts are a more accurate indication of trait impacts, because a reduction in the number of immatures indicates reduced oviposition in the previous generation. The relative ThryvOn reduction in the adult counts was variable between sites and years, and a consistent reduction of adults on ThryvOn was not observed. This is likely reflective of thrips adult movement and the lack of trait activity in the adults.

Unlike the adults, an overall impact of ThryvOn on thrips immatures was observed, except at some low-pressure sites in 2021. The variability at the low-pressure sites may be attributed to sampling variations that obscure the ThryvOn impact, which is suggested by the large within-site variations at these sites. When the number of samples was doubled in 2022, there was less within-site variations than in 2021, and a consistent impact of ThryvOn on thrips immatures was observed even at low-pressure sites. It is also possible that immigrants from the surrounding landscape had more of an impact on thrips populations in plots at low-pressure sites, making it more challenging to quantify the ThryvOn impact. Overall, the reduced within-site variation observed in 2022 compared to 2021 highlights the importance of focusing on the most informative observations (i.e., immature insect abundance) and increasing the sample size for these observations to decrease the variation and optimize the resistance monitoring approach.

As ThryvOn is a non-high-dose product and susceptible insects will be present, the baseline susceptibility data were presented as the percent reduction of immatures on ThryvOn relative to the control cotton at each site. In 2021, at moderate- to high-pressure locations, there was at least a 40% reduction in immatures on ThryvOn relative to the control, and in 2022, there was at least a 60% reduction of immatures on ThryvOn at all sites, regardless of the background insect pressure. These baseline susceptibility data can be used in the future within a resistance monitoring program to define unexpected injuries and/or less-than-expected control triggers that may indicate suspected resistance. One approach might be to estimate the underlying distribution of relative survival on ThryvOn (e.g., from the estimates in Figure 5), defining the trigger threshold as the 95th percentile of this distribution. In estimating the underlying distribution, less weight might be given to location estimates with greater confidence intervals, as in Efron and Morris (1977) [33].

ThryvOn will be deployed with additional IPM measures for thrips and the *Lygus* control, such as seed treatments and insecticidal oversprays [6,34], making it more difficult to detect changes in trait efficacy over time in commercial fields. This highlights the need to use sentinel plots for resistance monitoring in non-high-dose products going forward, where the impact of the trait can be assessed relative to the control and in the absence of insecticide applications (including seed treatments).

In conclusion, we determined that quantifying thrips immatures on ThryvOn relative to the control cotton was the best metric to characterize the baseline susceptibility data of thrips to ThryvOn cotton, and we optimized the sampling scheme for thrips immatures. These baseline data can be used in the future within a ThryvOn resistance monitoring program to identify unexpected injuries and potentially resistant populations. Additionally, this study demonstrates that resistance monitoring approaches can be optimized in non-high-dose systems by considering the key aspects of insect biology and interactions with the trait.

## Figures and Tables

**Table 1:** Academic cooperators and sentinel plot locations that generated 2021–2022 baseline susceptibility data for thrips species.

Site Code	Institution	Academic Cooperator(s)	Plot County/ Parish, State	Species
1AZ	University of Arizona	Peter Ellsworth	Pinal Co., AZ	*Frankliniella* *occidentalis*
2NC	North Carolina State University	Dominic Reisig	Washington Co., NC	*Frankliniella fusca*
3VA	Virginia Tech	Sally Taylor	Suffolk ^1^, VA	*Frankliniella fusca*
4MS	Mississippi State University	Angus Catchot	Tallahatchie Co., MS	*Frankliniella fusca*
5MS	Mississippi State University	Angus Catchot	Leflore Co., MS	*Frankliniella fusca*
6MS-A/6MS ^2^	Mississippi State University	Whitney Crow, Jeff Gore, Don Cook	Washington Co., MS	*Frankliniella fusca*
6MS-B ^3^	Mississippi State University	Whitney Crow, Jeff Gore, Don Cook	Coahoma Co., MS	*Frankliniella fusca*
7LA ^3,4^	Louisiana State University	Sebe Brown	Rapides Parish, LA	*Frankliniella fusca*
7TN ^4^	University of Tennessee	Sebe Brown	Madison Co., TN	*Frankliniella fusca*
8LA	Louisiana State University	Tyler Towles	Tensas Parish, LA	*Frankliniella fusca*
9AR ^3^	University of Arkansas	Gus Lorenz, Ben Thrash	Lee Co., AR	*Frankliniella fusca*

^1^ Suffolk is the closest municipality (no county) to the sentinel plot. ^2^ Represented as 6MS-A and 6MS in the 2021 and 2022 data, respectively. ^3^ 2021 only. ^4^ The 2021 Louisiana State University (Rapides Parish, LA, USA) site was replaced with a site at the University of Tennessee (Madison Co., TN, USA) in 2022.

## Data Availability

Data are available upon request.
